# Beyond BMI: a deeper dive into aortic aneurysm pathogenesis

**DOI:** 10.1097/JS9.0000000000003010

**Published:** 2025-07-08

**Authors:** Haipeng Li, Luyuan Chang, Jiaqi Yan

**Affiliations:** a Department of Mental Health, Bengbu Medical University, Bengbu, Anhui, China; b Department of Clinical Medicine, Bengbu Medical University, Bengbu, Anhui, China

*Dear Editor,*

We read with great interest the recent article by Jing and colleagues, “Global, regional, and national burden of aortic aneurysm attributable to body mass index from 1990 to 2021 and prediction to 2040: a cross-sectional study,” published in the *International Journal of Surgery*[1]. This comprehensive study, which integrates Global Burden of Disease data with UK Biobank and Mendelian randomization (MR) datasets, provides timely insights into the evolving landscape of aortic aneurysm (AA) burden attributable to high body mass index (BMI). However, we believe several key points warrant further elaboration. This study is compliant with the TITAN Guidelines 2025—governing declaration and use of AI[2].

Firstly, while the use of BMI is pragmatic in large-scale analyses, its limitations as a surrogate for adiposity are increasingly recognized. BMI fails to differentiate between subcutaneous and metabolically active visceral adipose tissue, which is more strongly implicated in AA pathogenesis. The “obesity paradox” in AA further challenges the clinical utility of BMI. A meta-analysis suggested a U-shaped association, with a nadir in mortality risk at a BMI of 28.55-31.05 kg/m^[2,3]^ The author also briefly mentioned that alternative indicators such as waist-to-hip ratio (WHR) and the novel weight-adjusted waist index offer superior discriminatory power for cardiovascular risk and better characterize fat and muscle composition. Notably, another MR study have established a positive causal relationship between WHR and AA risk[4].

Secondly, a more profound exploration of the molecular and cellular mechanisms linking adiposity to aortic wall degradation is crucial. Dysfunctional perivascular adipose tissue and adipokines actively contribute to inflammation and matrix remodeling in the aortic wall[5]. Matrix metalloproteinases and inflammatory cytokines are key drivers of extracellular matrix degradation, a hallmark of AA pathogenesis[6]. Pathological oxidative stress also plays a pivotal role, leading to vascular smooth muscle cell phenotypic switching and elastin/collagen degradation. These processes are modulated by sex hormones, genetic predispositions, and life-course exposures[7].

Finally, current projections often omit future medical advances or policy changes. Sophisticated dynamic models can integrate the impact of novel anti-obesity pharmacotherapies (GLP-1 receptor agonists) and large-scale public health interventions, providing a more nuanced understanding of future disease trajectories.

## Data Availability

All data are included in the article.
